# A Single Complex *Agpat2* Allele in a Patient With Partial Lipodystrophy

**DOI:** 10.3389/fphys.2018.01363

**Published:** 2018-09-26

**Authors:** Marjoleine F. Broekema, Maarten P. G. Massink, Joep De Ligt, Edwin C. A. Stigter, Houshang Monajemi, Jeroen De Ridder, Boudewijn M. T. Burgering, Gijs W. van Haaften, Eric Kalkhoven

**Affiliations:** ^1^Center for Molecular Medicine, University Medical Centre Utrecht, Utrecht University, Utrecht, Netherlands; ^2^Institute of Metabolic Science, Academic Medical Center, Amsterdam, Netherlands; ^3^Rijnstate Hospital, Arnhem, Netherlands

**Keywords:** lipodystrophy, AGPAT2, whole genome sequencing, adipose tissue, triacylglycerol synthesis, metabolic complications

## Abstract

Genetic lipodystrophies are a group of rare syndromes associated with major metabolic complications – including severe insulin resistance, type 2 diabetes mellitus, and hypertriglyceridemia – which are classified according to the distribution of adipose tissue. Lipodystrophies can be present at birth or develop during life and can range from local to partial and general. With at least 18 different genes implicated so far, definite diagnosis can be challenging due to clinical and genetic heterogeneity. In an adult female patient with clinical and metabolic features of partial lipodystrophy we identified via whole genome sequencing (WGS) a single complex *AGPAT2* allele [V67M;V167A], functionally equivalent to heterozygosity. *AGPAT2* encodes for an acyltransferase implicated in the biosynthesis of triacylglycerol and glycerophospholipids. So far homozygous and compound heterozygous mutations in *AGPAT2* have only been associated with generalized lipodystrophy. A SNP risk score analysis indicated that the index patient is not predisposed to lipodystrophy based on her genetic background. The partial phenotype in our patient is therefore more likely associated to the genetic variants in *AGPAT2.* To test whether the resulting double-mutant AGPAT2 protein is functional we analyzed its *in vitro* enzymatic activity via mass spectrometry. The resulting AGPAT2 double mutant is enzymatically inactive. Our data support the view that the current classification of lipodystrophies as strictly local, partial or generalized may have to be re-evaluated and viewed more as a continuum, both in terms of clinical presentation and underlying genetic causes. Better molecular understanding of lipodystrophies may lead to new therapies to treat adipose tissue dysfunction in common and rare diseases.

## Introduction

The absence and/or loss of adipose tissue is the hallmark of genetic lipodystrophies, a clinically and genetically heterogeneous group of extremely rare syndromes (Visser et al., 2011; Lightbourne and Brown, 2017). Genetic lipodystrophies are associated with severe metabolic complications, including severe insulin resistance, type 2 diabetes mellitus (T2DM), hypertriglyceridemia, and pancreatitis (Garg, 2011; Lightbourne and Brown, 2017). Initially, genetic lipodystrophies were classified according to the pattern of heritability and the distribution of the lost adipose tissue, which ranges from local to partial and general. With advances in molecular genetics, lipodystrophies were subclassified based on their genetic etiology. Two major categories can be distinguished: Familial partial lipodystrophy (FPLD) and congenital generalized lipodystrophy (CGL). FPLD is an autosomal dominant condition characterized by a lack of adipose tissue in the extremities with preservation/expansion of adipose tissue in face, trunk, and neck. Mutations in the genes *LMNA* and *PPARG* are the main genetic causes of FPLD (Garg, 2011). More than 300 patients with FPLD have been identified with *LMNA* mutations (OMIM 150330) and approximately 70 subjects harbor mutations in *PPARG* (OMIM 604367) (Garg, 2011). Patients with the autosomal recessive condition CGL often present in the neonatal period or early childhood with a generalized loss of adipose tissue. Since the first description of CGL, 300–500 patients have been reported worldwide (Garg, 2011). The majority of the patients with CGL (∼95%) harbors mutations in the genes *AGPAT2* (CGL1; OMIM 603100) and *BSCL2* (CGL2; OMIM 606158). At least 14 other genes have been implicated in genetic lipodystrophies (Patni and Garg, 2015). There is considerable clinical and genetic heterogeneity, making a definite diagnosis challenging (Patni and Garg, 2015).

Loss-of-function mutations in *AGPAT2* have been identified as the genetic cause of congenital generalized lipodystrophy type 1 (CGL1) (Agarwal et al., 2002). There is a lack of metabolically active adipose tissue, specifically at most of the subcutaneous areas, intra-abdominal regions, intra-thoracic regions, and bone marrow with preservation of mechanical adipose tissue depots including the orbits, palms, soles, and under the scalp (Patni and Garg, 2015). During childhood, patients develop severe insulin resistance and T2DM, hypertriglyceridemia, hepatic steatosis, and pancreatitis. Furthermore, patients manifest with accelerated growth and have an acromegaloid appearance, which is accompanied with voracious appetite due to leptin deficiency. Female patients often develop features of the polycystic ovary syndrome and successful pregnancy is rare. The patient characteristics of CGL1 are recapitulated in *Agpat2^−/−^* mice (Cortés et al., 2009; Vogel et al., 2011). Both male and female mice display a generalized lack of adipose tissue with metabolic complications such as insulin resistance, T2DM, hypertriglyceridemia, and hepatic steatosis.

**FIGURE 1:**
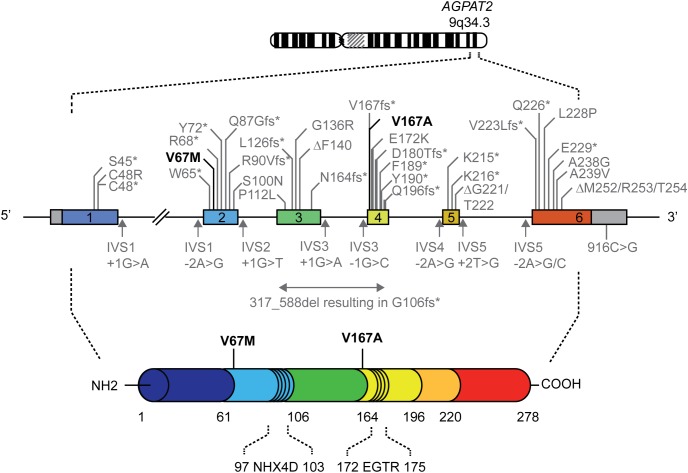
Genomic map of the *AGPAT2* gene showing mutations reported in patients with lipodystrophy. Numbered boxes represent exons and the in-between lines indicate introns. Missense, nonsense and frameshift mutations, and small deletions are indicated above the genomic map. Splice site and 3′-untranslated region mutations are shown below the genomic map. The mutations in bold are the mutations reported in the current study. Please note that the genomic map is not drawn to scale. A comprehensive overview of previously described *AGPAT2* mutations is provided in **Supplementary Table 1** (Agarwal et al., 2002, 2003; Magré et al., 2003; Fu et al., 2004; Gomes et al., 2004; Haque et al., 2005; Taleban et al., 2008; Miranda et al., 2009; Pelosini et al., 2011; Haghighi et al., 2012, 2016; Poovazhagi et al., 2013; Ramanathan et al., 2013; Cortés et al., 2014; Akinci et al., 2016; Shetty et al., 2016).

The high incidence of parental consanguinity and high recurrence rate in siblings suggest that CGL1 is an autosomal recessive disorder. Indeed, the majority of the patients harbor either homozygous or compound heterozygous *AGPAT2* mutations (**Figure 1** and **Supplementary Table 1**; Agarwal et al., 2002, 2003; Magré et al., 2003; Fu et al., 2004; Gomes et al., 2004; Haque et al., 2005; Taleban et al., 2008; Miranda et al., 2009; Pelosini et al., 2011; Haghighi et al., 2012, 2016; Poovazhagi et al., 2013; Ramanathan et al., 2013; Cortés et al., 2014; Akinci et al., 2016; Shetty et al., 2016). Noteworthy, individuals heterozygous for mutations in *AGPAT2* (e.g., parents and siblings of CGL1 patients) have no obvious CGL1 phenotype. However, they may suffer from more subtle metabolic derangements in glucose and lipid metabolism (Agarwal and Garg, 2006).

AGPAT2 is part of a gene family that has 11 members in humans (Agarwal, 2012). AGPAT2 is highly expressed in adipose tissue; the other AGPATs are also expressed in adipose tissue, albeit at lower levels (Agarwal, 2012). The *AGPAT2* gene is located on chromosome 9q34 and encompasses six exons that together encode a protein of 278 amino acids (Agarwal, 2012). *AGPAT2* encodes for 1-acyl-glycerol-3-phosphate-acyltransferase 2 (AGPAT2), an integral membrane protein of the endoplasmic reticulum, that adds a fatty acyl group to the sn-2 position of 1-acylglycerol-3-phosphate (lysophosphatidic acid; LPA), catalyzing the conversion to 1,2 diacylglycerol-3-phosphate (phosphatidic acid; PA) (Agarwal et al., 2002). PA is a key lipid intermediate in the biosynthesis of triacylglycerol and glycerophospholipids. The NHX_4_D and EGTR motifs are highly conserved among the AGPATs and both motifs are essential for enzymatic activity (Agarwal, 2012). It should be noted that the functional consequences of *AGPAT2* mutations on the enzymatic function of the protein has only been assessed in a limited number of cases (Haque et al., 2005; Taleban et al., 2008).

The molecular mechanisms underlying the generalized lack of adipose tissue due to mutations in *AGPAT2* are not exactly known. So far, only small deletions, nonsense, splice site, frameshift, missense, and 3′-untranslated region mutations in *AGPAT2* have been identified in patients affected by CGL1 (**Figure 1** and **Supplementary Table 1**). These mutations impair the enzymatic activity to a different extent, ranging from partially to almost completely (Patni and Garg, 2015). Subsequent dysregulation in the biosynthesis of triacylglycerol and glycerophospholipids is assumed to disrupt the integrity of adipocytes, leading to lipodystrophy. Alternatively, the regulatory role of AGPAT2 in PI3K/AKT and PPARγ signaling during adipogenesis might be disrupted (Subauste et al., 2012).

Here we report a single complex *AGPAT2* allele [V67M;V167A] in an adult female suffering from partial lipodystrophy by whole genome sequencing (WGS). The single complex allele generates an enzyme that is *in vitro* completely unable to incorporate a fatty acyl group into LPA. Taken together, our data support the use of WGS in atypical clinical cases and suggest that significant overlap exist, both in terms of clinical presentation and underlying genetic causes, between different types of lipodystrophies. Improved molecular understanding of lipodystrophies will contribute to development of new therapies to treat adipose tissue dysfunction in both common and rare diseases.

## Materials and Methods

### Case Presentation

Patient characteristics have been described before (Visser et al., 2011). Informed consent for participation and publication was obtained from the patient and the study was approved by the institutional review board of the Academic Medical Centre in Amsterdam, Netherlands (Visser et al., 2011). In short, the patient, a 58-year-old female from Southeast Asian descent, suffered from T2DM for which she was treated with 176 U/day. The insulin resistance was accompanied with acanthosis nigricans. With a BMI of 25 kg/m^2^, she furthermore experienced lipoatrophy in the extremities (skin fold thickness <25th percentile) and displayed a buffalo hump. Triglyceride level is 3.4 mmol/l (normal range 0.6–2.2 mmol/l). The partial aspect of the lipodystrophy in our patient was reflected by a leptin concentration of 27.7 ng/ml (normal range 3.7–11.1 ng/ml). Parents and siblings were not available for physical examination. Patient material was not available for additional exploratory analysis.

### Whole Genome Sequencing Analysis

Genomic DNA was extracted from peripheral-blood leukocytes in venous blood samples using QIAamp DNA Blood Mini Kit according to the manufacturer’s instructions (Qiagen Hilden, Germany). DNA libraries for Illumina sequencing were generated using standard protocols (Illumina) from 200 ng to 1 μg of genomic DNA. The libraries were sequenced with paired-end (2 × 150 bp) runs using Illumina HiSeq X Ten sequencers with a target depth of 30× base coverage. The Illumina data was processed with our in-house developed pipeline v 1.4.0^1^ including Genome Analysis Toolkit (GATK) v3.4-46 according to the best practices guidelines (McKenna et al., 2010; Van der Auwera et al., 2013). Briefly, sequence reads were mapped against the human reference genome GRCh37 using Burrows–Wheeler Aligner (BWA) v0.7.5a mapping tool (Li and Durbin, 2009). Sequence reads were marked for duplicates using Sambamba v0.5.8 and realigned per sample using GATK IndelRealigner (Tarasov et al., 2015). Next, GATK Haplotypecaller was used to call SNPs and indels to create gVCF’s. These gVCF’s were genotyped with GATK. Variants are flagged as PASS only if they do not meet the following criteria: QD < 2.0, MQ < 40.0, FS > 60.0, HaplotypeScore >13.0, MQRankSum <-12.5, ReadPosRankSum <-8.0, snpclusters ≥3 in 35 bp. For indels: QD < 2.0, FS > 200.0, ReadPosRankSum <-200. Variant Cartagenia Bench Lab NGS v.4.2.2 was used for variant interpretation and prioritization. The genetic variants were prioritized on the basis of the population frequency <0.5% in NHLBI GO Exome Sequencing Project (ESP) (2016), Genome Aggregation Database (gnomAD), 1000Genomes, and Single Nucleotide Polymorphism Database (dbSNP; accessed March 2017). Next, the putative pathogenic loss-of-function, splice-site and non-synonymous variants were assessed. After gene prioritizing we were left with two genetic variants in *AGPAT2.* The mutations identified in the patient, corresponds to c.199G > A/c.500T > C and p.V67M/p.V167A in reference NM_006412.3 and NP_006403.2, respectively. Raw genome sequence data from the index patient is available from the https://www.ebi.ac.uk/ega/home; Accession codes: EGAS00001003177 (study), EGAD00001004314 (dataset). Samples from parents or siblings could not be obtained for further genetic analysis. To test whether the identified genetic variants occurred in *cis* or *trans* configuration we performed PCR amplification of the genomic region encompassing exons 2–4 (Forward primer 5′ TGG ATG TGG ATT TGG AAG T 3′; reverse primer sequence 5′ GGA GGA GTC CCT TGT GTG TC 3′), cloned the fragment into pCR^TM^II-TOPO^®^ vector (Invitrogen) and sequenced 12 individual bacterial clones. Forty percent of the colonies were wildtype.

### Generation of Human AGPAT2 Mutant Constructs

A pCDNA3.1 vector containing the complete coding region of human AGPAT2 and C-terminal FLAG tag (Genscript) was used as a template to generate V67M, V167A, and V67M/V167A using the QuikChange mutagenesis kit (Stratagene) following the manufacturer’s instructions. Successful mutagenesis was verified by Sanger sequence analysis.

### AGPAT2 Activity Analysis by Mass Spectrometry

HEK293T human embryonic kidney cells were seeded in DMEM 4.5 g/L D-glucose supplemented with 10% fetal calf serum (Invitrogen), and 100 μg penicillin/ml and 100 μg streptomycin/ml (Invitrogen). The next day cells were transfected using X-tremeGENE 9 DNA Transfection Reagent according to manufacturer’s protocol with pCDNA3.1-AGPAT2 WT and mutant expression vectors. After 2 days cells were washed in ice-cold PBS on ice and harvested in lysis buffer (50 mM Tris–HCl pH 7.5, 250 mM NaCl, 5 mM EDTA, and 0.1% NP40) containing protease inhibitors (Complete, Roche Applied Biosciences). After rocking for 30′ at 4°C the cellular lysates were spun down at 16.100 × *g* for 5′ at 4°C. Protein concentrations in supernatant were measured using the Pierce BCA protein assay kit according to the manufacturer’s instructions. Cell lysates (200 μg of protein) were incubated with ANTI-FLAG^®^ M2 Affinity Agarose Gel (Sigma) for 2 h at 4°C. AGPAT2-coupled beads were washed three times with lysis buffer and subsequently washed in 100 mM Tris–HCl pH 7.5. The recombinant AGPAT2 acyltransferase activity was assayed using oleoyl-CoA (Sigma) as acyl donor and 1-(9Z-octadecenoyl)-sn-glycero-3-phosphate (LPA, Avanti Polar Lipids) as acyl acceptor. For this, FLAG-AGPAT2 WT or mutant coupled to beads was incubated for 30 min at 37°C in 100 mM Tris–HCl pH 7.5 containing 10 μM LPA, 50 μM oleoyl CoA, and 1 mg/ml BSA. All assays were performed in quadruple. The reactions were terminated by addition of chloroform:methanol (2:1). After thorough mixing the extracts were left at room temperature for 30 min. Extracts were centrifuged at 20.000 × *g* for 5 min for phase separation and the lower phase was transferred and evaporated. The residue was dissolved in 50% acetonitrile. The extracts were analyzed by liquid chromatography-mass spectrometry. Samples were analyzed using a Phenomenex EVO C18 column kept at 40°C and coupled to a Thermo Scientific LTQ Orbitrap-XL mass spectrometer operated in negative ion-mode. Upon injection a 10 min linear gradient was started from 0 to 100% eluent B (80% isopropanol 20% acetonitrile also containing 15 mM NH_4_OH) and kept at 100% B for 1 min followed by 100% eluent C (90% isopropanol 10% acetonitrile also containing 10 mM NH4Ac) for 5 min. The column was conditioned at 100% eluent A (10% acetonitrile containing 15 mM NH_4_OH) for 4 min prior to a next injection. Samples were kept at 8°C in the autosampler. The flow rate was 200 μL/min. The results are indicated as averages of the replicates ± SEM. Student’s *t*-tests were used. A statistically significant difference was defined as a *p*-value of <0.05.

### Western Blot Analysis

Cells and IP fractions were resolved in Laemmli sample buffer and subjected to SDS-PAGE and subsequently transferred to Immobilon membranes (Millipore). Anti-FLAG M2-HRP and anti-Tubulin were used for detection of the proteins. Enhanced chemiluminescence (Amersham Biosciences) was used for visualization.

### Association of Common Genetic Variants With Partial Lipodystrophic Phenotype

In the patient, WGS data was used to genotype lead SNPs in 53 genomic regions that were previously identified to be associated with a reduced ability to store adipose tissue in peripheral compartments (Lotta et al., 2017). Upon download of the full dataset SNPs (EGA accession EGAD00010000890) were extracted based on rsID from the imputed dataset (EGAS00001001232_UKHLS.UK10K+1KG-imputed.phwe_1e-4.info_0.4.filtered). These SNPs were further processed using plink (v. 1.9b3) to extract compound genotypes and dosage. A custom R script (code and data attached in supplement) was used to further process these data and calculate the number of risk alleles in the population and calculate a *z*-score.

## Results

### Identification of Two Genetic Variants in AGPAT2 in a Patient With Partial Lipodystrophy

The prevalence of genetic forms of lipodystrophy may be underestimated, as the phenotype can be heterogeneous and may subsequently not be recognized. We therefore previously screened a large diabetic patient database (*n* = 5,221) for marked insulin resistance (≥ 100 U insulin/day) and BMI ≤ 27 kg/m2, and identified five partial lipodystrophy cases (Visser et al., 2011). While one case may be explained by chronic corticoid steroid usage, in 3 cases a diagnosis could be made after targeted genetic analyses of the FPLD-associated genes *PPARG*, *LMNA*, and *CIDEC* (two *PPARG* mutations, one *LMNA* mutation) (Visser et al., 2011). However, no mutations were observed in these candidate genes in the last patient (referred to as S9, Visser et al., 2011). To identify the genetic cause underlying her lipodystrophic phenotype, we performed WGS. After filtering of the data we were left with two heterozygous genetic variants in exon 2 and exon 4 in *AGPAT2*, p.V67M and p.V167A, respectively (**Supplementary Figure 1**). These genetic variants were absent in the variation databases (all accessed in March 2017) (NHLBI GO Exome Sequencing Project (ESP), 2016) (Exome Variant Server), 1000Genomes (1000 Genomes Project Consortium, 2015), and Single Nucleotide Polymorphism Database (dbSNP) (Sherry et al., 2001). The Genome Aggregation Database (gnomAD, accessed in March 2017) (Lek et al., 2016) reveals an allele frequency of 0.01122 for p.V67M (9:139571992 C/T) and 0.0001949 for p.V167A (9:139571125 A/G), respectively, in the South Asian population (the patient is from Southeast Asian descent). Noteworthy, the gnomAD database contains exomes and genomes of individuals that are not necessarily healthy: Individuals with severe pediatric diseases have been removed, but many remaining individuals suffer from adult-onset diseases such as T2DM, a condition that is also associated with lipodystrophy (Lek et al., 2016). Although, a homozygous V67M mutation has been reported in a patient with a severe presentation of CGL1 previously (Poovazhagi et al., 2013), the V167A substitution has not been reported before. Various *in silico* prediction programs assessed V167A as deleterious (SIFT score = 0.004; FATHMM score = –3.52; Mutation Taster = disease causing).

**FIGURE 2:**
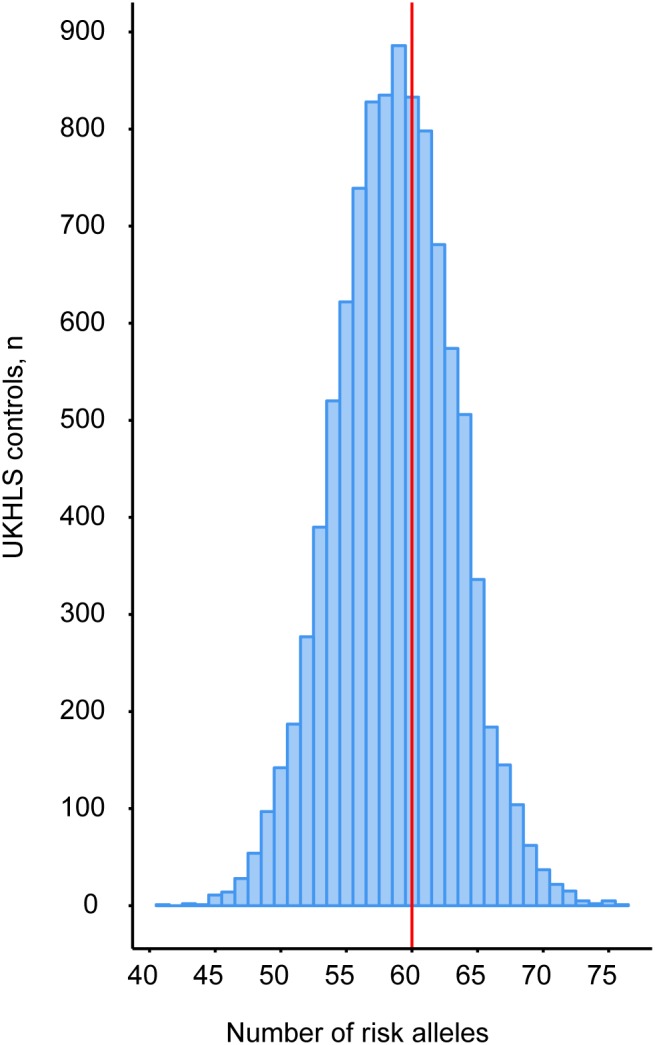
Peripheral adipose tissue expandability risk score analysis to determine the contribution of the patient’s genetic background to the partial lipodystrophic phenotype. Genotypes for these loci were extracted from the UKHLS GWAS dataset (EGA accession EGAD00010000890) to construct a background distribution in a healthy female population (blue bars). WGS revealed that our patient harbors 60 risk alleles (red vertical line).

A recent population-level genetic study not only reported that single nucleotide polymorphisms (SNPs) in 53 genomic regions are significantly associated with insulin resistance, but also that harboring an increased number of these risk alleles is accompanied with an impaired ability to expand adipose tissue in the peripheral compartments (Lotta et al., 2017). Patients with FPLD1, a subtype of partial lipodystrophy for which a genetic cause has not yet been identified, are significantly enriched for these SNPs (Lotta et al., 2017). This finding suggests a polygenic origin for this subtype of partial lipodystrophy. To explore whether the SNPs in these 53 loci contributed to the partial lipodystrophic phenotype in our patient we determined the number of risk alleles in our patient. To construct a background distribution, genotypes for these loci were also extracted from the female samples in the UKHLS GWAS dataset (EGA accession EGAD00010000890). Our patient harbors 60 risk alleles (**Figure 2** and **Supplementary Table 2**). Compared to this population distribution the index patient is not at increased risk (*z*-score = 0.27), suggesting that risk allele frequency is unlikely to be involved. Although, we cannot exclude genetic interactions, the partial phenotype in our patient is therefore more likely associated to the two identified genetic variants in *AGPAT2*. For this reason, these genetic variants were further characterized.

### Haplotype Analysis Reveals a Single Complex AGPAT2 Allele

**FIGURE 3:**
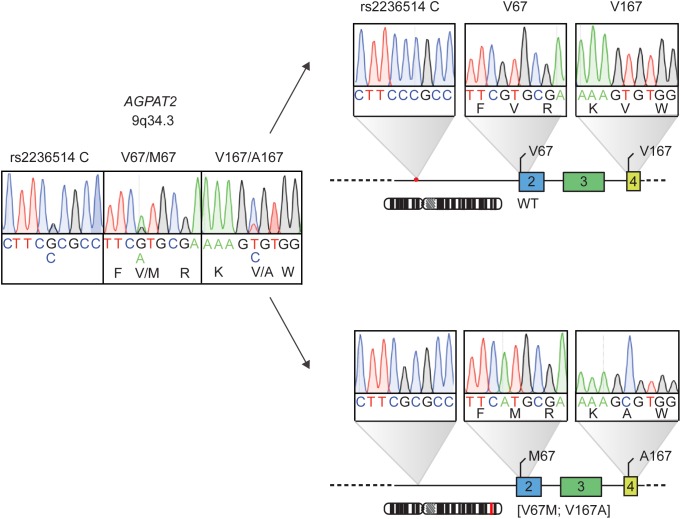
Haplotype analysis of the *AGPAT2* genetic variants identified by whole genome sequencing in patient with atypical partial lipodystrophy. The patient harbors two heterozygous mutations in *AGPAT2*, on the left. Haplotype analysis, on the right, shows a single complex *AGPAT2* allele [V67M;V167A] with the mutations situated in the *cis* configuration. The heterozygous C/G SNP rs2236514 causing the allele PCR bias is also shown.

The physical distance between the identified genetic variants was too large to determine via WGS whether the p.V67M and p.V167A occur in a *trans* configuration, meaning that the variants locate to the different alleles (functionally equivalent to homozygosity) or in the *cis* configuration, meaning that both variants affect one allele (functionally equivalent to heterozygosity). Samples from parents or siblings could not be obtained for further genetic analysis. The haplotype analysis in our patient indicated that she carries the two genetic variants in the *cis* configuration (**Figure 3**). Notably, the initial haplotype analysis was complicated by an allele amplification bias due to a heterozygous C/G SNP (rs2236514) upstream of exon 2 that juxtaposes the annealing site of the forward primer in the PCR, favoring the allele that harbors p.V67M. By using an intronic primer situated upstream from rs2236514 we were able to show that our patient is the first case of partial lipodystrophy associated with a single complex allele in *AGPAT2*.

### The Single Complex AGPAT2 Allele [V67M;V167A] Allele Generates a Complete Inactive Double Mutant AGPAT2 Enzyme

**FIGURE 4:**
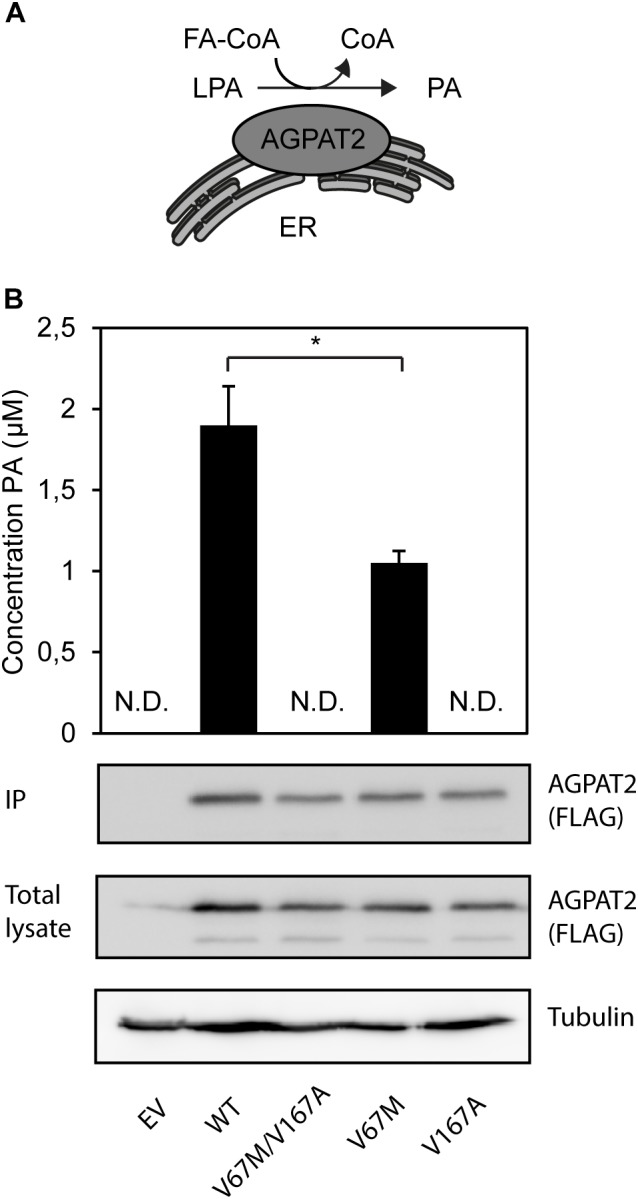
Enzymatic synthesis of PA by the AGPAT2 V67M/V167A double mutant. **(A)** The ER-integral membrane protein AGPAT2 catalyzes the conversion of LPA into PA. **(B)** PA is synthesized by immunopurified recombinant AGPAT2-FLAG by incorporating oleoyl-CoA into LPA. All assays were performed in quadruple. The results are indicated as averages of the replicates ± SEM. Student’s *t*-tests were used. ND, not determinable. A statistically significant difference was defined as a *p*-value of <0.05. Expression levels of AGPAT2-FLAG WT and mutant (IP and total cell lysate) was confirmed by western blotting using an antibody against FLAG.

The single complex *AGPAT2* allele [V67M;V167A] allele generates a double mutant AGPAT2 protein. The V167A amino acid residue is closely situated to the EGTR motif, which is a highly conserved motif essential for enzymatic activity (Agarwal, 2012). Patient-derived adipocytes were not available for additional analysis. To establish whether the enzymatic activity of the double-mutant AGPAT2 protein was affected, we developed an assay to monitor the *in vitro* conversion of LPA to PA by mass spectrometry (**Figure 4A**). The AGPAT2 V67M/V167A double mutant and the V167A single mutant are both completely enzymatically inactive, while the single mutant V67M retains 50% of the enzymatic activity (**Figure 4B**). These findings indicate that the V167A mutation is sufficient to cause a complete loss of activity in the double mutant AGPAT2 enzyme.

## Discussion

Here we report a single complex AGPAT2 allele [V67M;V167A], functionally equivalent to heterozygosity, in a patient with clinical and metabolic features of partial lipodystrophy; homozygous and compound heterozygous *AGPAT2* mutations have so far been associated with generalized lipodystrophy (Agarwal et al., 2002, 2003; Magré et al., 2003; Fu et al., 2004; Gomes et al., 2004; Haque et al., 2005; Taleban et al., 2008; Miranda et al., 2009; Pelosini et al., 2011; Haghighi et al., 2012, 2016; Poovazhagi et al., 2013; Ramanathan et al., 2013; Cortés et al., 2014; Akinci et al., 2016; Shetty et al., 2016). This single complex *APGAT2* allele renders a double-mutant protein that is enzymatically inactive. The clinical diagnosis of partial lipodystrophy, rather than generalized lipodystrophy, was based on the loss of adipose tissue mainly in the extremities and the leptin levels (27.7 ng/ml; normal range 3.7–11.1 ng/ml), which indicates the presence of significant amounts of functional adipose tissue (Visser et al., 2011). Our data suggest that there may be overlap between generalized and partial lipodystrophies, both in terms of clinical presentation and in underlying genetic causes. Compelling support for this view comes from a recent study showing that compound heterozygous mutations of *PPARG* – a gene previously only associated with partial lipodystrophy type 3 (FPLD3) – can cause CGL (Dyment et al., 2014).

Our initial haplotype analysis, in which exon 2 was PCR amplified with frequently used primers and analyzed by conventional Sanger sequencing, showed a clear amplification bias caused by a SNP upstream of exon 2. Hypothetically, the heterozygous C/G intronic SNP can also favor amplification of the wildtype allele, leading to a delay in genetic diagnosis. Moreover, it may be possible that other mutations in exon 2 that were reported previously in CGL1 cases are also heterozygous. Usage of next generation sequencing techniques (NGS) can minimize the number of genetic variants that may be missed by Sanger sequencing due to PCR amplification bias (Jéru et al., 2016).

In the current study, a patient with partial lipodystrophy was identified with a single complex *AGPAT2* allele, which is functionally equivalent to heterozygosity. So far, no obvious lipodystrophy (either generalized or partial) phenotypes have been described in individuals with heterozygous *AGPAT2* mutations. However, they may suffer from more subtle metabolic derangements (Agarwal and Garg, 2006). In agreement with this, heterozygous *Agpat2^−/+^* mice have no detectable lipodystrophy phenotype either (Vogel et al., 2011). Together, these findings suggest that additional genetic or other factors may be required to reduce the activity of the intact *AGPAT2* allele below a critical threshold level, resulting in a partial lipodystrophy phenotype. The importance of genetic background on the penetrance of lipodystrophy was recently illustrated in a large population-level genetic study in which 53 loci linked to an insulin resistance phenotype (i.e., high fasting insulin levels adjusted for BMI, low HDL cholesterol levels, and high triglyceride levels) were also associated with a limited peripheral adipose tissue expandability (Lotta et al., 2017). Patients with FPLD1, a more prevalent subtype of partial lipodystrophy with hitherto unknown genetic cause, were found to be significantly enriched for these SNPs, suggesting a polygenetic inheritance for FPLD1 (Lotta et al., 2017). This leads to the enticing hypothesis that a subtle impairment in adipose tissue expandability due to an enrichment of common variants can potentially contribute to the AGPAT2-associated partial lipodystrophic phenotype in our patient. The risk score analysis of the 53 genomic loci associated to peripheral adipose tissue expandability performed here suggests that the genetic background of our patient does not present an increased risk (*z*-score = 0.27). However, we cannot exclude genetic interactions between these loci or the contribution of additional genetic risk loci. Identification of additional heterozygous *AGPAT2* cases, within the rare group of subtle and partial lipodystrophies, is needed to investigate this further.

Based on our findings, we expect that application of WGS in cases of unexplained lipodystrophy will not only identify novel lipodystrophy-associated loci, but will also reveal more cases of heterozygous *AGPAT2* variants. This will further improve the molecular understanding of the clinical spectrum of lipodystrophies. In time, this may contribute to development of new therapeutic strategies to treat adipose tissue dysfunction in common and rare diseases.

## Author Contributions

MB performed the experiments, data-analysis, and wrote the manuscript. MM performed the whole genome sequencing analysis. JDL performed the SNP score risk analysis. ES contributed by developing, performing, and analyzing the mass spectrometry experiments. HM contributed to the clinical analysis. JDR advised on the SNP score risk analysis. BB provided guidance and advice to the study. GvH contributed in the whole genome sequencing analysis. EK designed and supervised the study. MB and EK wrote the manuscript. All authors reviewed the manuscript.

## Conflict of Interest Statement

The authors declare that the research was conducted in the absence of any commercial or financial relationships that could be construed as a potential conflict of interest.

## Footnotes

1 https://github.com/CuppenResearch/IAP
