# Supplementary material for: A Single Complex Agpat2 Allele in a Patient With Partial Lipodystrophy
Source: Front Physiol. 2018 Sep 26;9:1363. doi: 10.3389/fphys.2018.01363 (PMC6168662; doi:10.3389/fphys.2018.01363)
Supplement: Supplementary file 1 [file Data_Sheet_1.pdf]

A Single Complex *Agpat2* Allele In A Patient With Partial Lipodystrophy

Marjoleine F. Broekema[1], Maarten P.G. Massink[1], Joep De Ligt[1], Edwin C.A. Stigter[1], Houshang Monajemi[2,3], Jeroen De Ridder[1], Boudewijn M.T. Burgering[1], Gijs W. van Haaften[1], Eric Kalkhoven[1*].

[1] Center for Molecular Medicine, University Medical Centre Utrecht, Utrecht University, Utrecht, The Netherlands
[2] Institute of Metabolic Science, Academic Medical Center, Amsterdam, The Netherlands.
[3] Rijnstate Hospital, Arnhem, The Netherlands.

* Correspondence:
Dr. Eric Kalkhoven
e.kalkhoven@umcutrecht.nl

Running title: *AGPAT2* Affected In Partial Lipodystrophy

Supplementary Data

R script to determine the association of common genetic variants with partial lipodystrophic phenotype.

Supplementary Figure 1. Whole genome sequencing data showing the AGPAT2 variants in a patient with partial lipodystrophy. WGS reads of our patient aligned to the human reference genome GRCh37 (bottom). WGS results show that patient is heterozygous for rs2236514 and c.199G>A/c.500T>C, respectively. Please note that the reads are shown in reverse.

Supplementary Table 1. Overview of *AGPAT2* mutations in lipodystrophy. Overview of previously described mutations in *AGPAT2.* Genbank accession no. NM_006412.3 and NP_006403.2.# Originally described as c.636c>A. § Originally described as c.713A>T * Originally described as c.645 A>T

Supplementary Table 2. SNP genotype of patient with partial lipodystrophy. In the patient WGS data was used to genotype 53 genomic regions that were previously identified to be associated with a reduced ability to store adipose tissue in peripheral compartments (Lotta et al., 2017). We identified 60 risk alleles.

R script to determine the association of common genetic variants with partial lipodystrophic phenotype.

```
library("ggplot2")

setwd("53SNPs")

# Read relevant data

dosage <- read.table("UKHLS.dosage.txt.assoc.dosage",sep='',header=F, stringsAsFactors=F,
col.names=c("CHR","SNP","BP","A1","A2","FRQ","INFO","OR","SE","P"), skip=1 )

head(dosage)

gts <- read.table("plink.ped",sep=' ',header=F, stringsAsFactors=F)

head(gts[,1:10])

snpinfo <- read.table("SNPdata.txt",sep='\t',header=T, stringsAsFactors=F)

colnames(gts) <-
c("sample","family","pat","mat","gender","phenotype",as.character(snpinfo$SNP))

# Selecting females only

gts <- subset(gts, gender=="2")

# Determine where reference alle != non-risk allele

snpinfo$flip <- snpinfo$Effect.Allele!=dosage$A1

# Convert genotypes to risk scores

scores <- gts[,7:ncol(gts)]

scores[scores==0]<-NA

scores[scores==11]<-0

scores[scores==12]<-1

scores[scores==21]<-1

scores[scores==22]<-2

# Adjust risk scores for 'flipped' positions

scores[,snpinfo$SNP[snpinfo$flip]] <- scores[,snpinfo$SNP[snpinfo$flip]]-2

scores <- abs(scores)

# Determine missing genotypes to prevent over scoring

liponas <- apply(scores, 1, function(x) sum(is.na(x)))

liporisk <- rowSums(scores, na.rm=T)

liporiskfrac <- liporisk/((53-liponas)*2)

rownames(scores) <- gts$sample
```

```
scores$liporiskfrac <- liporiskfrac

scores$riskalleles <- scores$liporiskfrac*106

# Plot results of patient relative to population

pdf(file="LipoRiskDistribution_Paper.pdf",width=6,height=10,useDingbats=F, pointsize=10 )

aplot <- ggplot(scores, aes(x=riskalleles)) + geom_histogram(binwidth=1, fill="#4396F0", color="#4396F0", alpha=.5)

bplot <- aplot + ylab("UKHLS controls, n") + xlab("Number of risk alleles") + theme_bw() + theme(panel.border = element_blank(), panel.grid.major = element_blank(), panel.grid.minor = element_blank(), axis.line = element_line(colour = "black")) + scale_x_continuous(breaks=round(seq(40,80,by=5),1)) + scale_y_continuous(breaks=round(seq(0,900,by=100),1))

cplot <- bplot + theme(axis.text = element_text(size=15)) + theme(axis.title = element_text(size=20))

print(cplot + geom_vline(xintercept=53, color="red"))

dev.off()

# Calculate z-score

pop_sd <- sd(scores$riskalleles)*sqrt((length(scores$riskalleles)-1)/(length(scores$riskalleles)))

pop_mean <- mean(scores$riskalleles)

zscore <- (53 - pop_mean)/pop_sd

print(zscore)
```

Supplementary Figure 1: Whole genome sequencing data showing the AGPAT2 variants in a patient with partial lipodystrophy.

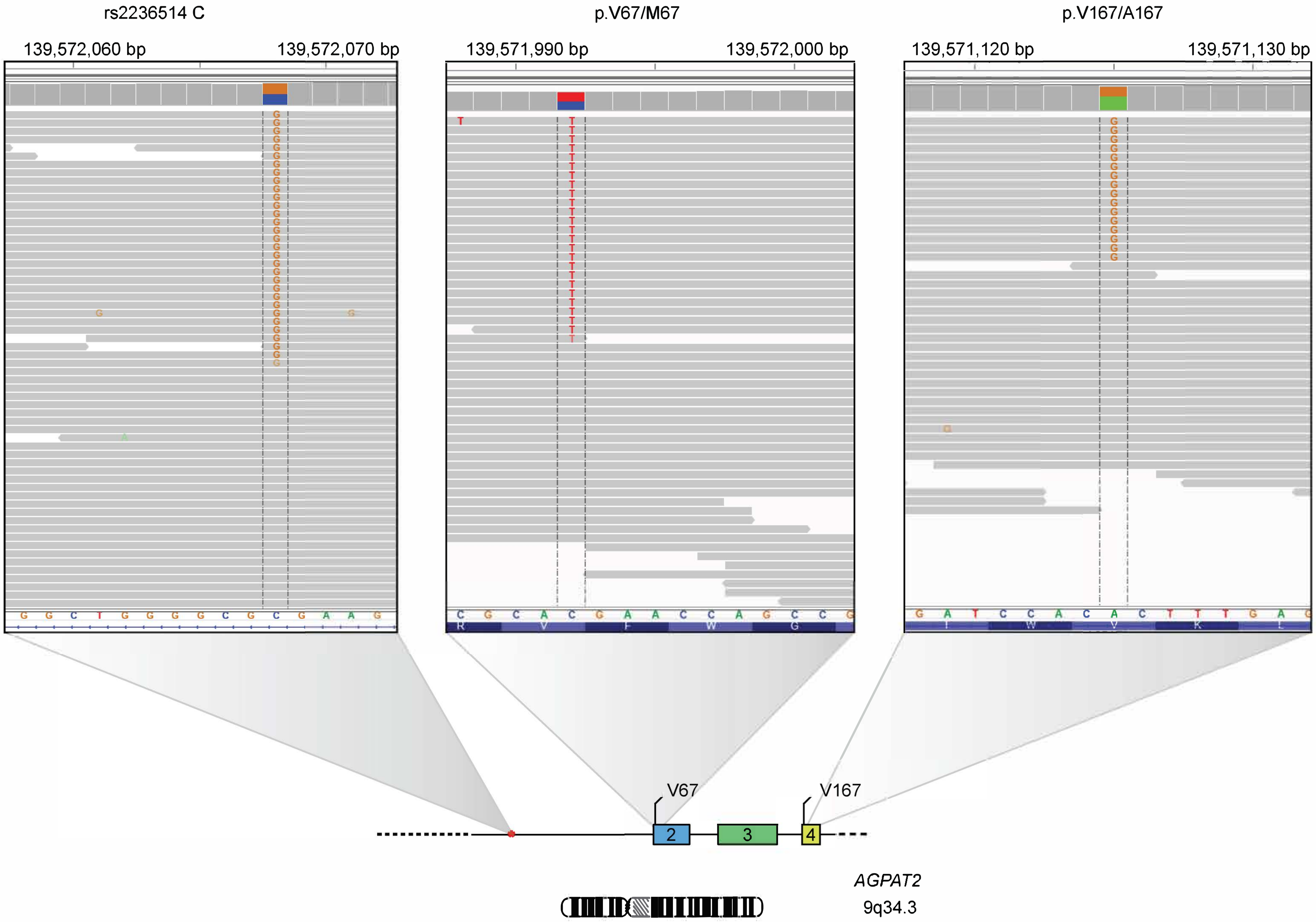

Supplementary Table 1. Overview of *AGPAT2* mutations in lipodystrophy.

	Protein	Genomic change	Location	Modes of inheritance	Reference
Missense	C48R	c.142T>C	Exon 1	Homozygous	(Ramanathan et al., 2013)
	V67M	c.199G>A	Exon 2	Homozygous/ single complex allele	(Poovazhagi et al., 2013; Current study)
	S100N	c.299G>A	Exon 2	Homozygous	(Agarwal et al., 2003; Cortés et al., 2014; Miranda et al., 2009)
	P112L	c.335C>T	Exon 2	Homozygous/ Compound heterozygous	(Pelosini et al., 2011; Taleban et al., 2008)
	G136R	c.406G>A	Exon 3	Compound heterozygous	(Agarwal et al., 2002)
	V167A	c.500T>C	Exon 4	Single complex allele	(Current study)
	E172K	c.514G>A	Exon 4	Homozygous	(Haghighi et al., 2012; Magré et al., 2003)
	L228P	c.683T>C	Exon 6	Compound heterozygous	(Agarwal et al., 2002)
	A238G	c.712C>G	Exon 6	Compound heterozygous	(Magré et al., 2003)
	A239V	c.716C>T	Exon 6	Compound heterozygous	(Agarwal et al., 2003)
Nonsense	S45X	c.134C>A	Exon 1	Homozygous	(Haghighi et al., 2016)
	C48X	c.144C>A	Exon 1	Homozygous	(Akinci et al., 2016)
	W65X	c.194G>A	Exon 2	Homozygous	(Magré et al., 2003)
	R68X	c.202C>T	Exon 2	Homozygous/ Compound heterozygous	(Agarwal et al., 2002; Akinci et al., 2016; Haghighi et al., 2016; Magré et al., 2003; Taleban et al., 2008)
	Y72X	c.216C>G	Exon 2	Homozygous	(Haghighi et al., 2016)
	F189X	c.567C>A[#]	Exon 4	Compound heterozygous	(Fu et al., 2004)
	Y190X	c.570C>A	Exon 4	Compound heterozygous	(Agarwal et al., 2003)
	K215X	c.645A>T[§]	Exon 5	Homozygous	(Fu et al., 2004)
	K216X	c.647A>T*	Exon 5	Homozygous	(Akinci et al., 2016; Magré et al., 2003)
	Q226X	c.676C>T	Exon 6	Homozygous	(Magré et al., 2003)
	E229X	c.685G>T	Exon 6	Homozygous	(Akinci et al., 2016; Haghighi et al., 2012)
Insertion	L126fsX	c.377insT	Exon 3	Compound heterozygous	(Agarwal et al., 2002)
	Q87GfsX	c.258_259insGGCTG	Exon 2	Homozygous	(Shetty et al., 2016)
Deletion	R90VfsX	c.268delC	Exon 2	Homozygous	(Akinci et al., 2016)
	140delF	c.418delTTC	Exon 3	Compound heterozygous	(Agarwal et al., 2002)
	V167fsX	c.504delGA	Exon 4	Compound heterozygous	(Agarwal et al., 2002)
	D180fsX	c.538delG	Exon 4	Compound heterozygous	(Agarwal et al., 2002; Haque et al., 2005)

	V223LfsX	c.667-705delinsCTGCG	Exon 6	Homozygous	(Akinci et al., 2016)
	252delMRT	c.755delTGAGGACCA	Exon 6	Homozygous	(Agarwal et al., 2002)
	G106fsX188	c.317-588del	Exon 2-4	Homozygous	(Agarwal et al., 2002; Fu et al., 2004; Gomes et al., 2004; Magré et al., 2003)
**Splice**		IVS1+1G>A	Intron 1	Compound heterozygous	(Agarwal et al., 2003)
	F60fsX102	IVS1-2A>G	Intron 1	Compound heterozygous	(Magré et al., 2003)
		IVS2+1G>T	Intron 2	Homozygous	(Akinci et al., 2016)
		IVS3+1G>A	Intron 3	Compound heterozygous	(Agarwal et al., 2003)
	N164fsX	IVS3-1G>C	Intron 3	Homozygous/ Compound heterozygous	(Agarwal et al., 2003; Cortés et al., 2014; Fu et al., 2004; Haque et al., 2005; Magré et al., 2003; Miranda et al., 2009)
	Q196fsX	IVS4-2A>G	Intron 4	Homozygous/ Compound heterozygous	(Agarwal et al., 2002, 2003; Fu et al., 2004; Haghighi et al., 2016; Magré et al., 2003)
		IVS5+2T>G	Intron 5	Homozygous	(Magré et al., 2003)
	221delGT	IVS5-2A>C	Intron 5	Homozygous	(Agarwal et al., 2002; Akinci et al., 2016)
**3'UTR**	c.916C>G		3'UTR	Compound heterozygous	(Agarwal et al., 2002)

Genbank accession no. NM_006412.3 and NP_006403.2.
# Originally described as c.636c>A.
§ Originally described as c.713A>T
* Originally described as c.645 A>T

References

Agarwal, A. K., Arioglu, E., De Almeida, S., Akkoc, N., Taylor, S. I., Bowcock, A. M., et al. (2002). AGPAT2 is mutated in congenital generalized lipodystrophy linked to chromosome 9q34. *Nature genetics* 31, 21–3. doi:10.1038/ng880.

Agarwal, A. K., Simha, V., Oral, E. A., Moran, S. A., Gorden, P., O'Rahilly, S., et al. (2003). Phenotypic and Genetic Heterogeneity in Congenital Generalized Lipodystrophy. *Journal of Clinical Endocrinology and Metabolism* 88, 4840–4847. doi:10.1210/jc.2003-030855.

Akinci, B., Onay, H., Demir, T., Ozen, S., Kayserili, H., Akinci, G., et al. (2016). Natural history of congenital generalized lipodystrophy: A nationwide study from Turkey. *Journal of Clinical Endocrinology and Metabolism* 101, 2759–2767. doi:10.1210/jc.2016-1005.

Cortés, V. A., Smalley, S. V., Goldenberg, D., Lagos, C. F., Hodgson, M. I., and Santos, J. L. (2014). Divergent metabolic phenotype between two sisters with congenital generalized lipodystrophy due to double AGPAT2 homozygous mutations. A clinical, genetic and in silico study. *PLoS ONE* 9, 1–6. doi:10.1371/journal.pone.0087173.

Fu, M., Kazlauskaite, R., Baracho, M. D. F. P., Do Nascimento Santos, M. G., Brandão-Neto, J., Villares, S., et al. (2004). Mutations in Gng3lg and AGPAT2 in Berardinelli-Seip congenital lipodystrophy and Brunzell syndrome: Phenotype variability suggests important modifier effects. *Journal of Clinical Endocrinology and Metabolism* 89, 2916–2922. doi:10.1210/jc.2003-030485.

Gomes, K. B., Fernandes, A. P., Ferreira, A. C. S., Pardini, H., Garg, A., Magré, J., et al. (2004). Mutations in the Seipin and AGPAT2 Genes Clustering in Consanguineous Families with Berardinelli-Seip Congenital Lipodystrophy from Two Separate Geographical Regions of Brazil. *The Journal of Clinical Endocrinology & Metabolism* 89, 357–361. Available at: http://dx.doi.org/10.1210/jc.2003-030415.

Haghighi, A., Kavehmanesh, Z., Haghighi, A., Salehzadeh, F., Santos-Simarro, F., Van Maldergem, L., et al. (2016). Congenital generalized lipodystrophy: Identification of novel variants and expansion of clinical spectrum. *Clinical Genetics* 89, 434–441. doi:10.1111/cge.12623.

Haghighi, A., Razzaghy-Azar, M., Talea, A., Sadeghian, M., Ellard, S., and Haghighi, A. (2012). Identification of a novel nonsense mutation and a missense substitution in the AGPAT2 gene causing congenital generalized lipodystrophy type 1. *European Journal of Medical Genetics* 55, 620–624. doi:10.1016/j.ejmg.2012.07.011.

Haque, W., Garg, A., and Agarwal, A. K. (2005). Enzymatic activity of naturally occurring 1-acylglycerol-3-phosphate-O- acyltransferase 2 mutants associated with congenital generalized lipodystrophy. *Biochemical and Biophysical Research Communications* 327, 446–453. doi:10.1016/j.bbrc.2004.12.024.

Magré, J., Delépine, M., Van Maldergem, L., Robert, J.-J., Maassen, J. A., Meier, M., et al. (2003). Prevalence of Mutations in AGPAT2 Among Human Lipodystrophies. *Diabetes* 52, 1573 LP-1578. Available at:

http://diabetes.diabetesjournals.org/content/52/6/1573.abstract.

Miranda, D. M., Wajchenberg, B. L., Calsolari, M. R., Aguiar, M. J., Silva, J. M. C. L., Ribeiro, M. G., et al. (2009). Novel mutations of the BSCL2 and AGPAT2 genes in 10 families with Berardinelli-Seip congenital generalized lipodystrophy syndrome. *Clinical Endocrinology* 71, 512–517. doi:10.1111/j.1365-2265.2009.03532.x.

Pelosini, C., Martinelli, S., Bagattini, B., Pucci, E., Fierabracci, P., Scartabelli, G., et al. (2011). Description of an AGPAT2 pathologic allelic variant in a 54-year-old Caucasian woman with Berardinelli-Seip syndrome. *Acta Diabetologica* 48, 243–246. doi:10.1007/s00592-011-0308-7.

Poovazhagi, V., Shanthi, S., Jahnavi, S., Radha, V., and Mohan, V. (2013). Berardinelli Seip congenital lipodystrophy presenting with neonatal diabetes mellitus due to a mutation in the AGPAT2 gene. *International Journal of Diabetes in Developing Countries* 33, 66–68. doi:10.1007/s13410-012-0099-6.

Ramanathan, N., Ahmed, M., Raffan, E., Stewart, C. L., O'Rahilly, S., Semple, R. K., et al. (2013). "Identification and Characterisation of a Novel Pathogenic Mutation in the Human Lipodystrophy Gene AGPAT2 BT - JIMD Reports - Case and Research Reports, 2012/6," in, eds. J. Zschocke, K. M. Gibson, G. Brown, E. Morava, and V. Peters (Berlin, Heidelberg: Springer Berlin Heidelberg), 73–80. doi:10.1007/8904_2012_181.

Shetty, S., Chapla, A., Kapoor, N., Thomas, N., and Paul, T. V. (2016). A novel variant of the AGPAT2 mutation in generalized congenital lipodystrophy , detected by next generation sequencing Implications for Practice : 9, 164–168.

Taleban, S., Carew, H. T., Dichek, H. L., Deeb, S. S., Hollenback, D., Weigle, D. S., et al. (2008). Energy balance in congenital generalized lipodystrophy type I. *Metabolism: Clinical and Experimental* 57, 1155–1161. doi:10.1016/j.metabol.2008.04.008.

Supplementary Table 2: SNP genotype of patient with atypical partial lipodystrophy.

SNP	Chromosome	Position	Effect Allele	Other Allele	Genotype	Risk Allele
rs683135	1	39895460	A	G	GG	0
rs17386142	1	50815783	C	T	CC	2
rs11577194	1	110500175	T	C	TT	2
rs9425291	1	172312769	A	G	GG	0
rs4846565	1	219722104	G	A	GG	2
rs2249105	2	65287896	A	G	AG	1
rs10195252	2	165513091	T	C	TT	2
rs492400	2	219349752	T	C	CT	1
rs2943645	2	227099180	T	C	CT	1
rs308971	3	12116620	G	A	AA	0
rs3864041	3	15185634	T	C	TT	2
rs295449	3	47375955	A	G	AA	2
rs11130329	3	52896855	A	C	AA	2
rs9881942	3	123082416	A	G	AG	1
rs645040	3	135926622	T	G	TT	2
rs2699429	4	3480136	C	T	TC	1
rs3822072	4	89741269	A	G	AA	2
rs6822892	4	157734675	A	G	GG	0
rs4865796	5	53272664	A	G	AA	2
rs459193	5	55806751	G	A	AA	0
rs4976033	5	67714246	G	A	AA	0
rs6887914	5	112711486	C	T	CT	1
rs1045241	5	118729286	C	T	CT	1
rs2434612	5	158022041	G	A	AA	0
rs966544	5	173350405	G	A	AA	0
rs12525532	6	35004819	T	C	CC	0
rs6937438	6	43815364	A	G	AA	2
rs2745353	6	127452935	T	C	CT	1
rs9492443	6	130398731	C	T	CC	2
rs3861397	6	139828916	G	A	AA	0
rs17169104	7	15883727	G	C	CG	1
rs972283	7	130466854	G	A	AA	0
rs2126259	8	9185146	T	C	CC	0
rs1011685	8	19830769	C	T	CC	2
rs4738141	8	72469742	G	A	AA	0
rs7005992	8	126528955	C	G	CC	2
rs498313	9	78034169	A	G	AA	2
rs10995441	10	64869239	G	T	GT	1
rs11231693	11	63862612	A	G	GG	0
rs17402950	12	14571671	G	A	AA	0
rs718314	12	26453283	G	A	AG	1
rs7973683	12	124449223	C	A	CC	2
rs7323406	13	111628195	A	G	GG	0
rs7176058	15	39464167	A	G	AA	2
rs8032586	15	73081067	C	T	CC	2
rs754814	17	4657034	T	C	TT	2
rs7227237	18	47174679	C	T	CC	2
rs8101064	19	7293119	T	C	TC	1
rs4804833	19	7970635	A	G	AG	1
rs4804311	19	8615589	A	G	AA	2
rs731839	19	33899065	G	A	GG	2
rs6066149	20	45602638	G	A	GG	2
rs132985	22	38563471	C	T	CT	1
